# The Contribution of Cytomegalovirus Infection to Immune Senescence Is Set by the Infectious Dose

**DOI:** 10.3389/fimmu.2017.01953

**Published:** 2018-01-10

**Authors:** Anke Redeker, Ester B. M. Remmerswaal, Esmé T. I. van der Gracht, Suzanne P. M. Welten, Thomas Höllt, Frits Koning, Luka Cicin-Sain, Janko Nikolich-Žugich, Ineke J. M. ten Berge, René A. W. van Lier, Vincent van Unen, Ramon Arens

**Affiliations:** ^1^Department of Immunohematology and Blood Transfusion, Leiden University Medical Center, Leiden, Netherlands; ^2^Department of Experimental Immunology, Academic Medical Center, Amsterdam, Netherlands; ^3^Renal Transplant Unit, Division of Internal Medicine, Academic Medical Center, Amsterdam, Netherlands; ^4^Delft University of Technology, Delft, Netherlands; ^5^Helmholtz Centre for Infection Research, Braunschweig, Germany; ^6^Department of Immunobiology, University of Arizona College of Medicine, Tucson, AZ, United States; ^7^Sanquin Blood Supply Foundation and Landsteiner Laboratory, Amsterdam, Netherlands

**Keywords:** CMV, memory CD8 T cells, immunesenesence, mouse models, memory inflation

## Abstract

The relationship between human cytomegalovirus (HCMV) infections and accelerated immune senescence is controversial. Whereas some studies reported a CMV-associated impaired capacity to control heterologous infections at old age, other studies could not confirm this. We hypothesized that these discrepancies might relate to the variability in the infectious dose of CMV occurring in real life. Here, we investigated the influence of persistent CMV infection on immune perturbations and specifically addressed the role of the infectious dose on the contribution of CMV to accelerated immune senescence. We show in experimental mouse models that the degree of mouse CMV (MCMV)-specific memory CD8^+^ T cell accumulation and the phenotypic T cell profile are directly influenced by the infectious dose, and data on HCMV-specific T cells indicate a similar connection. Detailed cluster analysis of the memory CD8^+^ T cell development showed that high-dose infection causes a differentiation pathway that progresses faster throughout the life span of the host, suggesting a virus–host balance that is influenced by aging and infectious dose. Importantly, short-term MCMV infection in adult mice is not disadvantageous for heterologous superinfection with lymphocytic choriomeningitis virus (LCMV). However, following long-term CMV infection the strength of the CD8^+^ T cell immunity to LCMV superinfection was affected by the initial CMV infectious dose, wherein a high infectious dose was found to be a prerequisite for impaired heterologous immunity. Altogether our results underscore the importance of stratification based on the size and differentiation of the CMV-specific memory T cell pools for the impact on immune senescence, and indicate that reduction of the latent/lytic viral load can be beneficial to diminish CMV-associated immune senescence.

## Introduction

Age-related decline in immunological competence, often referred to as immune senescence, is associated with an increased incidence of cancer, infections, and a reduced efficacy of vaccines (1). Factors thought to contribute to this progressive decline of immune responses include genetic, molecular, and cellular defects, but also environmental stressors such as chronic infections.

Human cytomegalovirus (HCMV), a member of the betaherpesvirus family, persists in the majority of the adult population (2). Compared to other infections, extraordinary high memory T cell frequencies, on average ~10% of the total memory T cell compartment, are noticed in peripheral blood of healthy individuals (3). Especially at old age, it has been observed that the HCMV-specific memory T cell pool can even occupy up to 50% of the total memory compartment (4). This phenomenon of high frequencies of circulating memory T cells that are maintained during the lifespan of the host or even can undergo gradual increment, aptly named memory inflation (5), is characteristic of CMV infection in general, as this is also found upon infection with the related mouse CMV (MCMV) and Rhesus Macaque CMV (RhCMV) [reviewed in Ref. (6, 7)]. The inflationary T cells, which increase in numbers during the persistent phase of infection, are phenotypically characterized by an “effector-memory (EM)”-like appearance (CD27^low^/CD62L^low^/CD127^−^/KLRG1^+^; e.g., the CD8^+^ T cell response against the m139_419–426_, M38_316–323_, IE3_416–423_ epitopes in MCMV). During CMV infection, T cells with a central-memory phenotype also exist (CD27^high^/CD62L^high^/CD127^+^), which peak early during infection, contract, and establish stable memory pools (e.g., the CD8^+^ T cell response against the M45_985–993_ epitope in MCMV). The occurrence of inflationary T cell responses is reliant on the intermittent transcription of CMV during latency resulting in antigen reactivation (8). In addition, the efficacy of peptide processing by the constitutive proteasome is an important determinant for memory inflation (9).

Epidemiological studies have shown an association between HCMV positivity and manifestations of immune senescence by linking persistent HCMV infection to decreased overall survival in the elderly and to the immune risk profile (IRP), a cluster of parameters predictive for 2-year mortality in the elderly (10, 11). In addition, anti-HCMV IgG titers in aged individuals have been correlated with lower antibody responses to influenza (12–15); however, other studies could not confirm this finding (16–19). Thus, whether HCMV infection is causally linked with accelerated immune senescence is a topic of debate (20, 21).

To gain more insight into the possible connection between CMV infection and immune senescence, mouse studies have been performed previously in which it was demonstrated that long-term CMV infection impairs newly generated CD8^+^ T cell responses to heterologous infections (22–24). However, these studies have been conducted using relatively high doses of virus, and have not taken into account the variability in real life with respect to the infectious dose. For example, in the human population the percentages of CMV-specific T cells occupying the memory T cell compartment is highly variable (ranging from 0.01 to 50%) (3), which is likely related to the significant difference in the quantity of CMV found in bodily fluids, such as breast milk, saliva, and urine (10^1^–10^5^ copies/μl), causing horizontal transmission of CMV (25, 26). In this respect, we have previously shown that the degree of accumulation and phenotype of inflationary CMV-specific CD8^+^ T cells is corresponding to the size of the initial infectious dose (27).

We anticipate that the viral inoculum size is highly variable between individuals and that this accounts for the large variance in the frequency, phenotype, and accumulation of CMV-specific CD8^+^ memory T cells in infected humans, which may be an explanation for the controversial results with respect to the possible contribution of CMV to immune senescence. Here, we tested this hypothesis in a highly controlled prospective study using the mouse model of CMV infection, which mimics CMV infection in humans (28). We inoculated mice with different inoculum dosages of MCMV and investigated longitudinally the influence of lifelong CMV infection on alterations within the peripheral T cell pool. To specifically assess the role of the CMV inoculum size on the development of heterologous anti-viral immunity, aged MCMV-infected mice received a challenge with lymphocytic choriomeningitis virus (LCMV). Our results show that the viral inoculum size determines the degree of CMV-induced immune alterations in lifelong infection. Furthermore, we demonstrate that only infection caused by a high MCMV dose reduces newly generated CD8^+^ T cell responses to heterologous superinfection. To our knowledge, this is the first evidence that the inoculum size of CMV is a crucial determinant for the development of CMV-associated impaired immunity in aging.

## Materials and Methods

### Mice

Wild-type (WT) C57BL/6 mice were purchased from Charles River. All mice were maintained under specific pathogen-free conditions at the Central Animal Facility of LUMC. Mice were housed under 12 h light/dark cycle, feed *ad libitum* and were 7–10 weeks old at the beginning of each experiment.

### Viruses

Mouse CMV-Smith was obtained from the American Type Culture Collection (ATCC VR-194; Manassas, VA, USA) and salivary gland stocks were prepared from infected BALB/c mice. WT mice matched for gender and age were infected i.p. with indicated dosages of salivary gland derived MCMV-Smith. For weekly infections with MCMV mice received 5 × 10^4^ PFU MCMV weekly for 1 year. Vaccinia virus expressing IE1 of MCMV (VACV-IE1) was produced as described elsewhere (29). BALB/c × DBA/2 F1 mice were infected with 1 × 10^6^ PFU (VACV-IE1) as described (23). LCMV-Armstrong was propagated on BHK cells and titers of virus stocks and organ homogenates were determined by plaque assays on Vero cells as described. For LCMV-Armstrong infection, WT mice (uninfected and previously infected with MCMV) were infected i.p. with 2 × 10^5^ PFU. LCMV titers in the lungs and kidneys were determined by a virus focus forming assay on Vero 76 cells as described elsewhere (30).

### Study Subjects

For phenotypical analysis of HCMV-specific T cell responses, PBMCs from HCMV-seropositive healthy donors and from initially HCMV-seronegative recipients (HLA-A*0101^+^, HLA-A*0201^+^, HLA-B*0702^+^, HLA-B*3501^+^) receiving a HCMV-positive kidney transplant were isolated and labeled for flow cytometry analysis (31). Quantitative PCR for HCMV was performed in EDTA-treated whole-blood samples, as described elsewhere (32).

### Flow Cytometry

MHC class I tetramer staining combined with phenotyping, and intracellular cytokine staining were performed to determine the magnitude and characteristics of the mouse viral-specific T cell responses as described (33). Single-cell suspensions were prepared from spleens obtained from uninfected and infected mice by mincing the tissue through a 70-µm cell strainer (BD Bioscience). Blood was collected from the tail vein. Erythrocytes were lysed in a hypotonic ammonium chloride buffer. Fluorochrome-conjugated antibodies specific for mouse CD3, CD4, CD8, CD27, CD44, CD62L, CD127 (IL-7Rα), IFN-γ, IL-2, KLRG1, and TNF were purchased from BD Biosciences, Biolegend, or eBioscience. Analysis of human PBMCs was performed as described (31). Fluorochrome-conjugated antibodies specific for human CCR7, CD3, CD8, CD27, CD28, CD45RA, CD57, CD127, and KLRG1 were purchased from BD Biosciences, Biolegend, or eBioscience. Cells were acquired using a BD LSR Fortessa flow cytometer, and data were analyzed using FlowJo software (TreeStar) and Cytosplore (34). Dead cells were excluded using live/dead markers. Gating strategies were performed as described (27, 31).

### MHC Class I Tetramers and Synthetic Peptides

The following class I-restricted peptides were used: M45_985–993_, m139_419–426_, M38_316–323_, IE3_416–423_, IE1_168–176_ (MCMV), GP33_33–41_, NP_396–404_, GP_276–286_ (LCMV). A pool of the following class II-restricted MCMV peptides were used: M09_133–147_, M25_409–423_, m139_560–574_, and m142_24–38_ (35). The following class II-restricted LCMV peptide was used: GP_61–80_. APC and PE-labeled MHC class I tetrameric complexes with the above-described peptide epitopes were used. For analysis of HCMV-specific CD8^+^ T cell responses, MHC class I tetrameric complexes with the following peptides were used: pp65_363–373_ (HLA-A*0101), pp65_495–503_ (HLA-A*0201), pp65_417–426_ (HLA-B*0702), pp65_123–131_ (HLA-B*3501).

### Multiplex

Blood was collected retro-orbitally and clotted for 30 min. After centrifugation, serum was collected and stored at −80°C until further use. Cytokines were measured in serum using a mouse Bio-Plex Pro Mouse Cytokine 23-plex immunoassay (Bio-Rad, Herculus, CA, USA) according to manufacturer’s protocol.

### Serum Antibody Detection by ELISA

Total IgM and IgG concentrations were determined by ELISA in serum samples as described earlier (27). Briefly, Nunc-Immuno Maxisorp plates (Fisher Scientific) were coated overnight with virus in bicarbonate buffer, and after blocking (skim milk powder, Fluka BioChemika), sera from mice were added. Next, plates were incubated with various HRP-conjugated antibodies (SouthernBiotech) to detect IgM/IgG. Plates were developed with TMB substrate (Sigma Aldrich), and the color reaction was stopped by the addition of 1 M H_2_SO_4_. Optical density was read at 450 nm (OD_450_) using a Microplate reader (Model 680, Bio-Rad).

### Statistical Analysis

To determine statistical significance between two groups, an unpaired Student’s *t*-test was performed. Significance between more than two groups was evaluated by one-way ANOVA and Tukey’s *post hoc* test was performed to correct for multiple comparisons. Mann–Whitney *U* test and Kruskal-Wallis test were performed to determine statistical differences in the viral load in mice. To test the strength of the linear relationship between the frequency of EM CD8^+^ T cells and the LCMV viral load, Pearson correlation was used. For correlations between the frequency of EM CD8^+^ T cells and the magnitude of the response, Spearman correlation was used. GraphPad Prism 6.0 software (GraphPad Software, La Jolla, CA, USA) was used for statistical analyses.

## Results

### Disparate Effects of CMV Infection and Aging on Naive and Memory CD8^+^ and CD4^+^ T Cell Subsets

**Figure 1:**
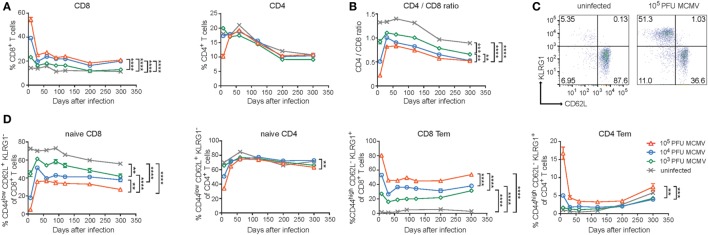
Disparate effects of CMV infection and aging on naive and memory CD8^+^ and CD4^+^ T cell subsets. Wild-type (WT) mice were infected with 10^3^, 10^4^, or 10^5^ PFU mouse CMV (MCMV)-Smith. **(A)** The average frequencies of CD8^+^ and CD4^+^ T cells in blood determined at the indicated time points post-infection. **(B)** The CD4^+^/CD8^+^ T cell ratio in blood determined at the indicated time points. **(C)** Representative flow cytometry plots showing the KLRG1 versus CD62L expression on CD8^+^ T cells of naive and 10^5^ PFU MCMV-infected mice. **(D)** Kinetic analysis of the average frequencies of naive (CD44^low^CD62L^+^KLRG1^−^) or effector-memory (Tem) (CD44^high^CD62L^−^KLRG1^+^) CD8^+^ and CD4^+^ T cells in blood. Error bars indicate SEM (*n* = 16 mice per group). Significance is indicated for day 300 post-infection. (*P < 0.05; **P < 0.01; ***P < 0.001; ****P < 0.0001). Data were pooled from two independent experiments.

To investigate whether dissimilar infectious dosages of MCMV differentially affect the immune system in lifelong infection, we inoculated C57BL/6 mice at 7 weeks of age with different dosages of MCMV-Smith (0, 10^3^, 10^4^, and 10^5^ PFU) and monitored the CD4^+^ and CD8^+^ T cell frequencies over 300 days post-infection (dpi). In naive (uninfected) mice, the frequency of total peripheral CD8^+^ T cells was unchanged in time. However, early after MCMV infection (acute phase), CD8^+^ T cell frequencies were elevated corresponding to the inoculum size (Figure 1A). A strict impact of the inoculum size was also observed at later time points: mice that were infected with the highest MCMV PFU quantities showed the highest frequency of CD8^+^ T cells. Only mice infected with the lowest inoculum dose (10^3^ PFU) did not differ substantially during the persistent phase from naive age-matched controls (Figure 1A). The frequency of total peripheral CD4^+^ T cells in naive and infected mice showed an age-related decrease, yet effects of the dosage of MCMV infection were not apparent for this T cell subset (Figure 1A).

It has been suggested that HCMV-seropositivity is linked to the IRP and one of the parameters defining the IRP is an inverted CD4/CD8 T cell ratio (36). Moreover, it has been shown that latent MCMV infection decreases this ratio (23). By calculating, the CD4/CD8 T cell ratio, we also observed a decreased CD4/CD8 T cell ratio during the persistent phase and markedly noticed an obvious MCMV-dose impact for this phenomenon (Figure 1B). A decreased CD4/CD8 T cell ratio was also most apparent in high-dose-infected aged mice based on the absolute counts of CD8^+^ and CD4^+^ T cells present in the spleen, and this was mainly caused by an increment of the absolute CD8^+^ T cell count (Figure S1A in Supplementary Material).

Next, we assessed the influence of the different MCMV inoculum sizes on the naive and memory T cell subset composition during the course of infection. MCMV infection resulted in strongly reduced frequencies of naive circulating CD8^+^ T cells (CD44^low^CD62L^+^KLRG1^−^), which inversely correlated with the inoculum dose (Figures 1C,D). Aging reduced the naive CD8^+^ T cell frequencies in both uninfected and infected mice. The differences between the groups of differently MCMV dosage inoculated mice remained over time. The naive CD4^+^ T cell pool was not overtly influenced by the inoculum size or aging (Figure 1D).

Accumulation of EM (CD44^high^CD62L^−^KLRG1^+^) CD8^+^ T cells was not observed in uninfected animals. However, this was clearly present after MCMV infection. Strikingly, increasing inoculum sizes correspondingly heightened the frequency of EM CD8^+^ T cells. These differences in EM CD8^+^ T cell frequency between the groups remained stable during persistent infection and somewhat inclined after 200 dpi (Figure 1D). The EM CD4^+^ T cell frequencies were increased after the highest dose inoculum and remained higher compared to lower dose infected mice and uninfected mice (Figure 1D). Aging increased the EM CD4^+^ T cell frequency in both infected and uninfected mice after 200 dpi. Thus, the size of the MCMV inoculum predominantly affects the formation of EM-like CD8^+^ T cells throughout the course of infection while aging seems to predominantly impact the percentages of EM-like CD4^+^ T cells.

### The Magnitude and Phenotype of Inflationary MCMV-Specific CD8^+^ T Cells Is Strongly Influenced by the Inoculum Dose

**Figure 2:**
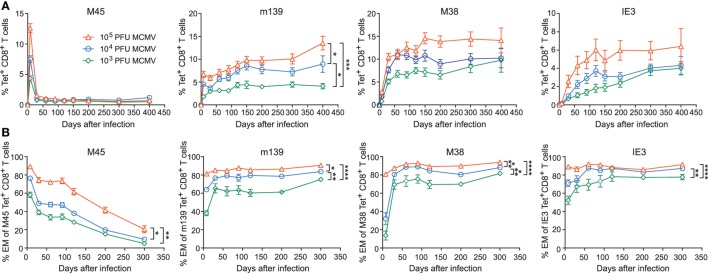
The magnitude and phenotype of inflationary mouse CMV (MCMV)-specific CD8^+^ T cells is strongly influenced by the inoculum dose. Wild-type (WT) mice were infected with 10^3^, 10^4^, or 10^5^ PFU MCMV-Smith. **(A)** The average frequencies of MCMV-specific CD8^+^ T cells (i.e., specific to epitopes derived from the MCMV proteins M45, m139, M38, and IE3) in blood were determined by MHC class I tetramer staining at the indicated time points post-infection. Error bars indicate SEM (*n* = 16 mice per group). **(B)** Kinetic analysis of the average frequencies of EM (CD44^high^CD62L^−^KLRG1^+^) type CD8^+^ T cells within the total MCMV-specific CD8^+^ T cell population in blood. Error bars indicate SEM (*n* = 16 mice per group). Significance is indicated for day 300 post-infection. (*P < 0.05; **P < 0.01; ***P < 0.001; ****P < 0.0001). Data were pooled from two independent experiments.

Next, we determined if dissimilarities in inoculum dosage sizes would differentially affect MCMV-specific CD8^+^ T cell populations during aging. In the acute phase of infection, we observed that the magnitude of both non-inflationary (M45-specific) and inflationary (m139-, M38-, and IE3-specific) MCMV-specific CD8^+^ T cell responses were correspondingly ~2-fold higher in the group infected with a 10-fold higher dose. During the chronic phase of infection, the frequency of the M45-specific CD8^+^ T cells is comparable between the different inoculum sizes. By contrast, the magnitude of the CD8^+^ T cell response against the inflationary epitopes in m139, M38, and IE3 increased in a strict dose-dependent manner (Figure 2A).

Induction of MCMV-associated immune senescence may be potentiated by the accumulation of virus-specific EM-like CD8^+^ T cells. Here, we assessed the phenotype of the antigen-specific CD8^+^ T cells that were induced after inoculation with the different MCMV doses. For all MCMV-specific CD8^+^ T cell responses measured, we observed that the size of the viral inoculum determines the frequency of effector CD8^+^ T cells during the acute phase (Figure 2B). While the effector phenotypic profile of the non-inflationary M45-specific T cells is gradually turned into a predominant central-memory (CM; CD44^high^CD62L^+^ KLRG1^−^) phenotype, the CD8^+^ T cells that are specific for the inflationary epitopes exhibit an increased effector-type appearance even after the acute phase of infection (Figure 2B; Figure S1B in Supplementary Material). The percentage of inflationary MCMV-specific CM CD8^+^ T cells is low throughout infection (Figure S1C in Supplementary Material). Thus, the initial infectious dose has a dominant influence and shapes both the magnitude and phenotype of inflationary MCMV-specific CD8^+^ T cells.

### Analogous Correlations between the Magnitude and Phenotype of Inflationary Mouse and Human CMV-Specific CD8^+^ T Cells

**Figure 3:**
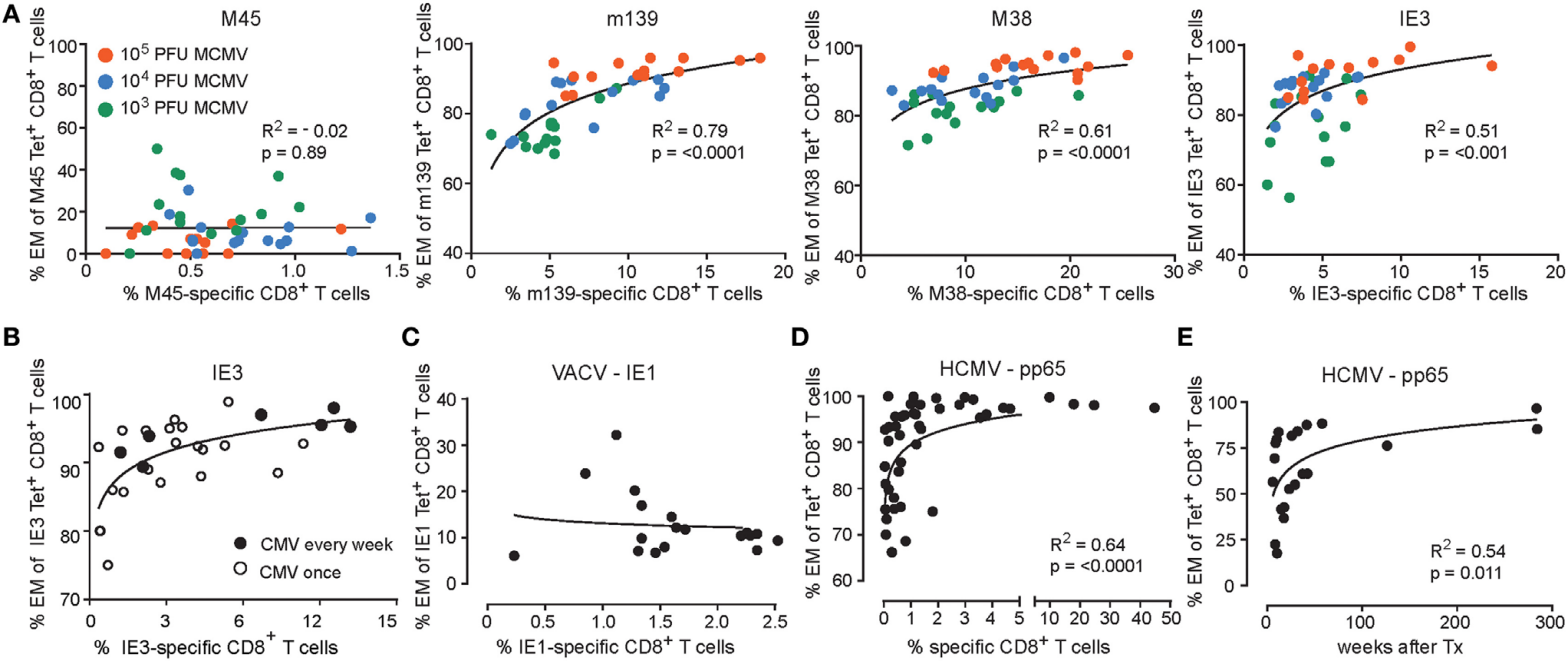
Correlations between the magnitude and phenotype of inflationary CMV-specific T cells. **(A)** Wild-type (WT) mice were infected with 10^3^, 10^4^ or 10^5^ PFU mouse CMV (MCMV)-Smith. The percentage of MCMV-specific EM (CD44^high^CD62L^−^KLRG1^+^) CD8^+^ T cells versus the percentage of MCMV-specific CD8^+^ T cells in blood at day 300 post-infection. Data were pooled from two independent experiments. **(B)** WT mice were infected with 5 × 10^4^ PFU MCMV-Smith once (*n* = 20) or weekly (*n* = 7) for 1 year. At 1 year post-infection, the average frequencies and phenotype of IE3-specific CD8^+^ T cells in blood was determined by MHC class I tetramer staining. The graph shows the percentage of IE3-specific EM (CD44^high^CD62L^−^KLRG1^+^) CD8^+^ T cells versus the percentage of IE3-specific CD8^+^ T cells in blood. **(C)** WT mice were infected with recombinant vaccinia virus expressing IE1 (VACV-IE1). At day 200 post-infection, the average frequencies and phenotype of IE1-specific CD8^+^ T cells in blood was determined by MHC class I tetramer staining. The graph shows the percentage of IE1-specific EM (CD44^high^CD62L^−^KLRG1^+^) CD8^+^ T cells versus the percentage of IE1-specific CD8^+^ T cells in blood (*n* = 18). **(D)** Analysis of human cytomegalovirus (HCMV)-specific CD8^+^ T cell responses. The percentage of antigen-specific EM CD8^+^ T cells versus the percentage of antigen-specific CD8^+^ T cells in blood from healthy donors for HCMV pp65 (*n* = 46). **(E)** The percentage of antigen-specific EM CD8^+^ T in blood from initially HCMV-seronegative recipients receiving a HCMV-positive kidney transplant against the weeks after transplantation.

Subsequently, we evaluated whether correlations exist between the magnitude and phenotype of the CMV-specific CD8^+^ T cells. Strikingly, only in case of responses against inflationary epitopes, a direct correlation between the magnitude of the MCMV-specific CD8^+^ T cell response and the frequency of the EM-like cells within the MCMV-specific CD8^+^ T cell population is observed (Figure 3). In mice infected with the lowest dose (i.e., 1 × 10^3^ PFU), the magnitude of the CD8^+^ T cell response against the inflationary epitopes and the frequency of effector cells is correspondingly the lowest, whereas these are highest in infection with the highest dose (i.e., 1 × 10^5^ PFU) (Figure 3A). Also, when we inoculated mice weekly with the same dose of MCMV for a period of 1 year, we found these correlations between the magnitude and phenotype of the inflationary CD8^+^ T cells, and these correlations were similar to those found in mice infected with MCMV once (Figure 3B). Responses against IE1 in the context of MCMV infection results in a similar correlation as observed for IE3 (data not shown) while IE1 expressed by recombinant vaccinia virus (VACV-IE1), eliciting acute but not persistent infection, did not elicit EM phenotype versus magnitude correlations (Figure 3C), Moreover, the IE1-specific CD8^+^ T cell response elicited by VACV-IE1 is with respect to the magnitude and EM kinetics similar to the M45-specific response elicited by high-dose MCMV [(23) and data not shown]. Together, these results indicate that viral antigen reactivation is essential for the observed correlations between the magnitude and EM phenotype of the CD8^+^ T cells.

Since humans are likely infected with varying doses of HCMV, we aimed to recapitulate whether the above-described correlations also exist in HCMV-positive individuals. Therefore, we performed analysis of the human pp65-specific CD8^+^ T cell response in a large cohort of healthy HCMV-seropositive individuals. Essentially, reminiscent to the correlations of the inflationary responses observed in the mouse (Figure 3A), a relationship was observed between the magnitude of the HCMV-specific CD8^+^ T cell response and the frequency of specific CD8^+^ T cells harboring an EM phenotype (Figure 3D). Remarkably, the development of the EM phenotype of CD8^+^ T cells against HCMV pp65 follows analogous kinetics upon primary infection of HCMV-seronegative patients receiving a HCMV-positive kidney transplant as compared to the EM phenotype of the inflationary MCMV epitopes (Figures 2B and 3E). Together, these data indicate that CD8^+^ T cell responses against viral epitopes in HCMV and MCMV follow a similar developmental pathway in which the EM phenotype of the responding CD8^+^ T cells correlate with the magnitude of the viral-specific CD8^+^ T cell response.

### Progressive EM CD8^+^ T Cell Differentiation Is Accelerated by High-Dose CMV Infection

**Figure 4:**
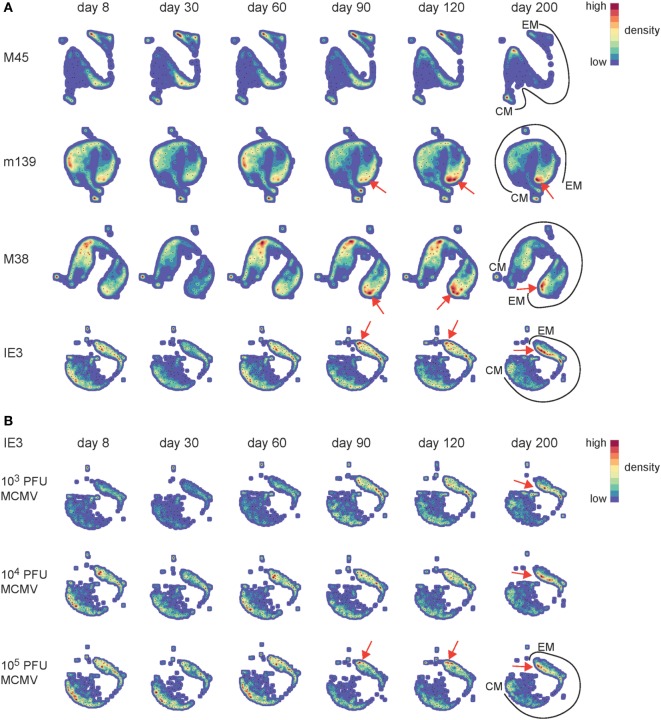
Progressive effector-memory (EM) CD8^+^ T cell differentiation is accelerated by high-dose CMV infection. Wild-type (WT) mice were infected with 10^3^, 10^4^, or 10^5^ PFU mouse CMV (MCMV)-Smith. MCMV-specific CD8^+^ T cells in blood were stained with MHC class I tetramers combined with cell surface markers (CD62L, KLRG1, CD27, and CD44) at the indicated time points post-infection. **(A)** Cytosplore analysis of the MCMV-specific CD8^+^ T cells in time. A-tSNE plots depict the pooled phenotypical data of MCMV-specific CD8^+^ T cells of the 10^3^, 10^4^, and 10^5^ PFU MCMV-infected mice for each time point after infection. **(B)** A-tSNE plots depict the phenotype of the IE3-specific CD8^+^ T cell response of the 10^3^, 10^4^, and 10^5^ PFU MCMV-infected mice for each time point after infection (*n* = 16 mice per MCMV dose). Data were pooled from two independent experiments. In the A-tSNE plots, the differentiation path from the CM and EM phenotype is specified. The red arrows indicate the ongoing shift toward a higher advanced EM phenotype.

To gain a more detailed understanding of the phenotypic differences of the MCMV-specific CD8^+^ T cells that develop during the low, intermediate, and high-dose infection, we used a novel analysis platform, termed Cytosplore (34), for immune cell phenotyping that incorporates approximated t-distributed stochastic neighborhood embedding (A-tSNE) for dimensionality reduction and—the mean—shift clustering algorithm for subset definition, based on the dimensionality reduced data (37). The A-tSNE algorithm lays out cells in a two-dimensional scatter plot, based on similarity of defined markers. Similar cells will be placed closed together in the plot, while slight variations in the level of marker expression will result in gradual positional transitions. The results of the A-tSNE algorithm are then visualized as density plots. This provides an unprecedented insight into the development of the cellular differentiation, which is not feasible by conventional flow cytometry data analysis. We performed Cytosplore analysis on the MCMV-specific CD8^+^ T cell populations we tracked in time by MHC class I tetramer staining combined with phenotypical markers comprising CD62L, KLRG1, CD27, and CD44, which allow discrimination between CM- and EM-type T cells (Figure S2A in Supplementary Material). Such analysis should display the potential differentiation trajectory path of EM- and CM-type T cells. In order to provide an initial global picture of the impact of CMV infection on the EM/CM T cell differentiation, we analyzed each MCMV-specific CD8^+^ T cell population in time using the combined data from the different infection dosages. We observed that the development of the EM phenotype of the inflationary T cells is progressive in time, as evidenced by an ongoing shift toward a higher advanced EM phenotype (indicated with red arrows) (Figure 4A). Even between days 120 and 200, the EM differentiation continues. To assess the role of dosing in this phenomenon, we performed the analysis with the separate dosages. Compared to low-dose infection, higher dose infections accelerated the EM CD8^+^ T cell differentiation throughout the infection (Figure 4B, shown for IE3; Figure S2B in Supplementary Material, shown for M38). We also observed that the impact of the dosage effect on the EM CD8^+^ T cell differentiation is irrespective of the infection duration (Figure S3 in Supplementary Material). Together these data suggest that high-dose infection causes an EM CD8^+^ T cell differentiation that not only develops faster but also continues to segregate throughout the life span of the host, suggesting a virus–host equilibrium that is influenced by aging and the infectious dose.

### The Dichotomy in Cytokine Polyfunctionality of Inflationary versus Non-Inflationary CMV-Specific T cells Increases with the Infectious Dose

**Figure 5:**
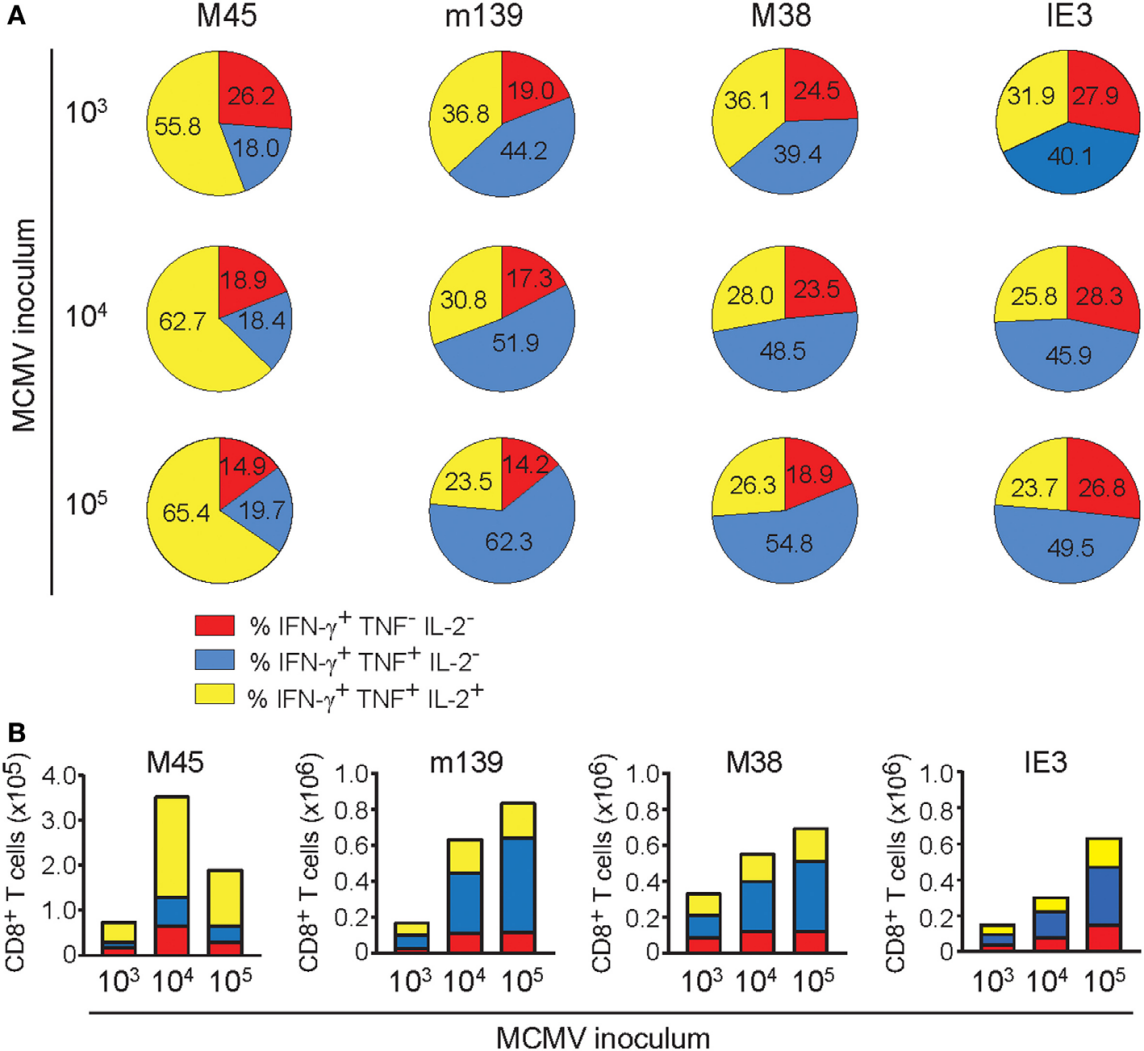
The dichotomy in cytokine polyfunctionality of inflationary versus non-inflationary CMV-specific T cells increases with the infectious dose. Following mouse CMV (MCMV) infection with different dosages (10^3^, 10^4^, or 10^5^ PFU MCMV-Smith), the cytokine polyfunctionality of splenic CD8^+^ T cells was determined after peptide restimulation at day 400 post-infection. **(A)** Pie charts depict the percentages of the single (IFN-γ), double (IFN-γ/TNF), and triple (IFN-γ/TNF/IL-2) cytokine producers of each antigen-specific T cell population upon peptide stimulation. **(B)** Absolute counts of the single (IFN-γ), double (IFN-γ/TNF), and triple (IFN-γ/TNF/IL-2) cytokine producers of each antigen-specific T cell population upon peptide stimulation. Data represents mean values (*n* = 8 per group). Data were pooled from two independent experiments.

In elderly individuals, a higher frequency of cytokine-deficient CD8^+^ T cells recognizing the HCMV epitope pp65 is associated with all-cause mortality in these individuals, suggesting that the presence of these cells could predict age-related dysfunction and increased risk of death (38). To gain insight into the cytokine profile of the MCMV-specific T cells in differentially inoculated aged mice, we assessed the polyfunctional cytokine profiles 400 days after MCMV infection. The majority of the M45-specific CD8^+^ T cell pool in the spleen comprises the capacity to coproduce IFN-γ, TNF, and IL-2 (Figure 5A). The frequency of these triple cytokine producers is elevated in mice infected with higher inoculums of MCMV. By contrast, CD8^+^ T cell populations specific for the inflationary epitopes (m139, M38, and IE3), consist most frequently of double-producing cells (IFN-γ and TNF). These frequencies increase with higher MCMV inoculum dosages, while this coincides with decreasing frequencies of triple cytokine producing cells (Figure 5A). Correspondingly, high-dose infection results in elevated absolute numbers of double IFN-γ/TNF producers, especially among the inflationary CD8^+^ T cells (Figure 5B). In the MCMV-specific CD4^+^ T cell pool a dose-dependent increase of double cytokine producers is also observed, although mainly at the expense of the single IFN-γ-producing cells (Figure S4A in Supplementary Material). Thus, whereas non-inflationary CD8^+^ T cells have an increment of the most polyfunctional T cells with higher inoculums, inflationary CD8^+^ T cells tend to lose this property.

### Only High-Dose CMV Infection Impairs the Development of Heterologous T Cell Responses

**Figure 6:**
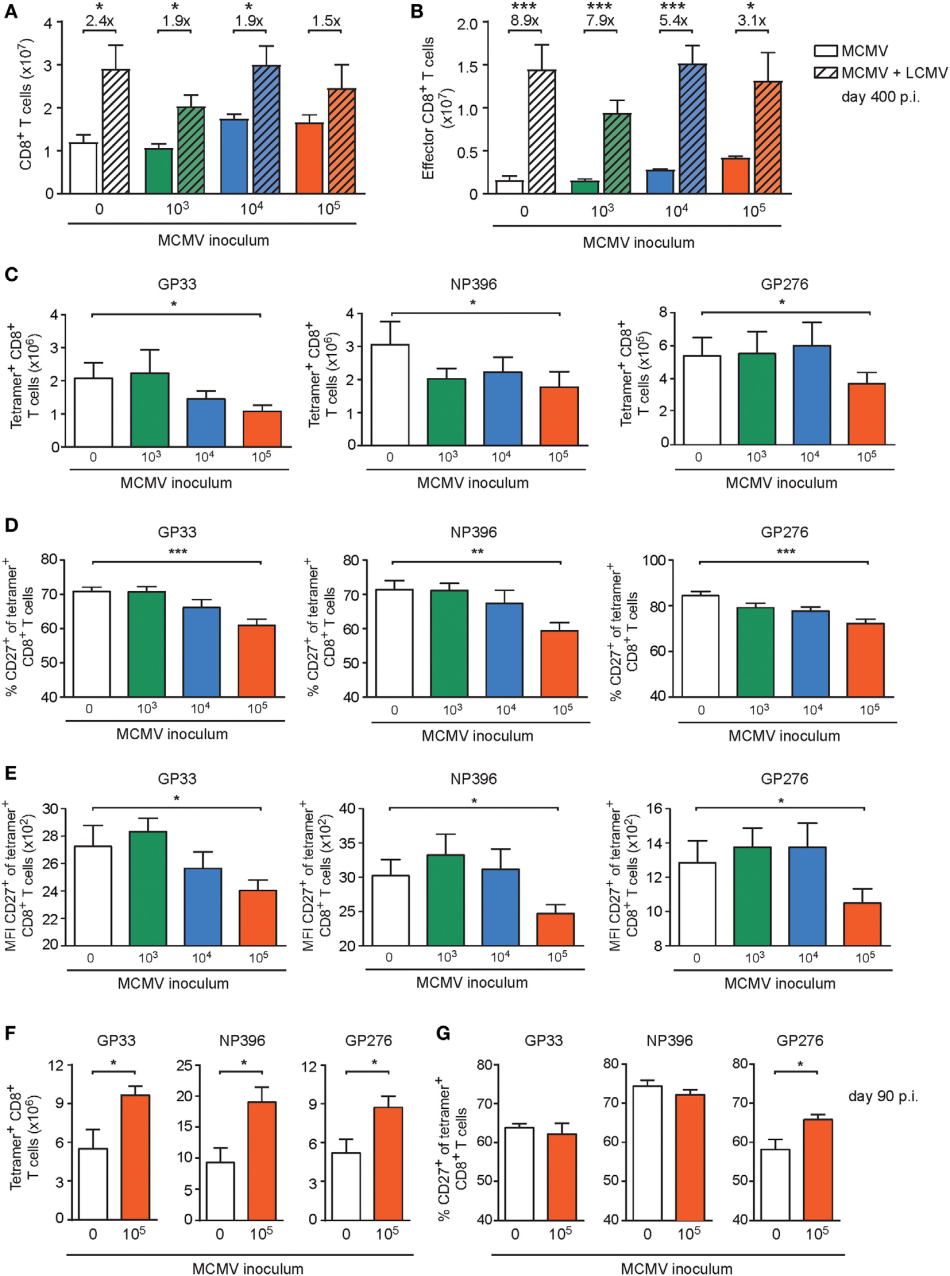
High-dose CMV infection impairs the development of heterologous viral-specific T cell responses. **(A–E)** Wild-type (WT) mice were kept uninfected or infected with 10^3^, 10^4^, or 10^5^ PFU mouse CMV (MCMV)-Smith (*n* = 16 mice per group), and at day 400 post-infection 8 mice per group were challenged with 2 × 10^5^ PFU lymphocytic choriomeningitis virus (LCMV)-Armstrong. At day 8 post LCMV infection, mice were analyzed and compared with uninfected littermate controls. **(A)** Numbers of total splenic CD8^+^ T cells. **(B)** Numbers of total effector (CD44^high^CD62L^−^ KLRG1^+^) CD8^+^ T cells. **(C)** Numbers of splenic LCMV-specific CD8^+^ T cells determined by MHC class I tetramer staining. **(D)** Percentage of LCMV-specific CD8^+^ T cells expressing CD27. **(E)** Mean fluorescence intensity (MFI) of CD27 expression on LCMV-specific CD8^+^ T cells. **(F,G)** WT mice were kept uninfected or infected with or 10^5^ PFU MCMV-Smith (*n* = 8 mice per group), and at day 90 post-infection challenged with 2 × 10^5^ PFU LCMV-Armstrong. At day 8 post LCMV infection, mice were analyzed and compared with uninfected littermate controls. **(F)** Numbers of splenic LCMV-specific CD8^+^ T cells determined by MHC class I tetramer staining. **(G)** Percentage of LCMV-specific CD8^+^ T cells expressing CD27. Results are represented as means and error bars indicate SEM (*n* = 8 mice per group). *P < 0.05; **P < 0.01; ***P < 0.001. Data were pooled from two independent experiments.

To assess whether the initial infectious dosages that determine the degree of the CMV-induced perturbations of peripheral naive and memory T cell compartments is connected to differential impairment of the development of heterologous anti-viral immunity, we challenged 400-day-old naive and MCMV-infected mice (1 × 10^3^, 1 × 10^4^, or 1 × 10^5^ PFU MCMV) with 2 × 10^5^ PFU LCMV-Armstrong. LCMV infection significantly increased the absolute splenic CD8^+^ T cell numbers of the naïve mice and of the mice infected with 1 × 10^3^ or 1 × 10^4^ PFU MCMV compared to age-matched mice that did not receive a LCMV challenge (Figure 6A). Strikingly, in mice that were long-term infected with the highest dose of MCMV (1 × 10^5^ PFU), the LCMV-induced CD8^+^ T cell expansion was impaired and numbers were not significantly increased (Figure 6A). Determination of the effector CD8^+^ T cell quantities revealed a similar trend; albeit LCMV challenge caused a significant increase in all groups, the expansion was least pronounced when mice were previously infected with the highest dose of MCMV (Figure 6B), suggesting a reduction in LCMV-specific CD8^+^ T cells when mice experienced high-dose MCMV inoculums previously. To test this assumption, we determined absolute numbers of LCMV-specific (GP33_33–41_, NP_396–404_, and GP_276–286_) CD8^+^ T cells by MHC class I tetramer binding (Figure 6C), and found indeed that the response against these immunodominant LCMV epitopes was mostly reduced in mice that were inoculated with the highest dose of MCMV (1 × 10^5^ PFU). Analysis of the LCMV-specific CD4^+^ T cell response (GP_61–80_) as measured by IFN-γ production revealed no MCMV-induced perturbations (Figures S4B,C in Supplementary Material). Together, these data suggest that high-dose MCMV infection affects the capacity of immunodominant CD8^+^ T cells to expand upon heterologous infections.

To assess whether the increasing doses of MCMV could differentially affect the activation status of the LCMV-primed CD8^+^ T cells, the cell surface expression of CD27, CD127, CD62L, and KLRG1 was evaluated. Strikingly, we found that the frequency of the immunodominant LCMV-specific CD8^+^ T cells expressing CD27, and correspondingly the expression of CD27 on a per cell basis, was mostly reduced in mice persistently infected with the highest MCMV dose (Figures 6D,E), while expression of CD127, CD62L, and KLRG1 was not influenced by MCMV infection (Figures S5A–C in Supplementary Material). Next, we investigated if the impairment of heterologous immune responses due to high-dose CMV infection is related to long-term infection of aged mice. Strikingly, the LCMV-specific CD8^+^ T cell response in young mice (5 months old) that were previously uninfected or infected with a high-dose MCMV (1 × 10^5^ PFU MCMV-Smith) revealed no reduction or even an increase in the amount or activation status of LCMV-specific CD8^+^ T cells (Figures 6F,G; Figure S5D–G in Supplementary Material).

**Figure 7:**
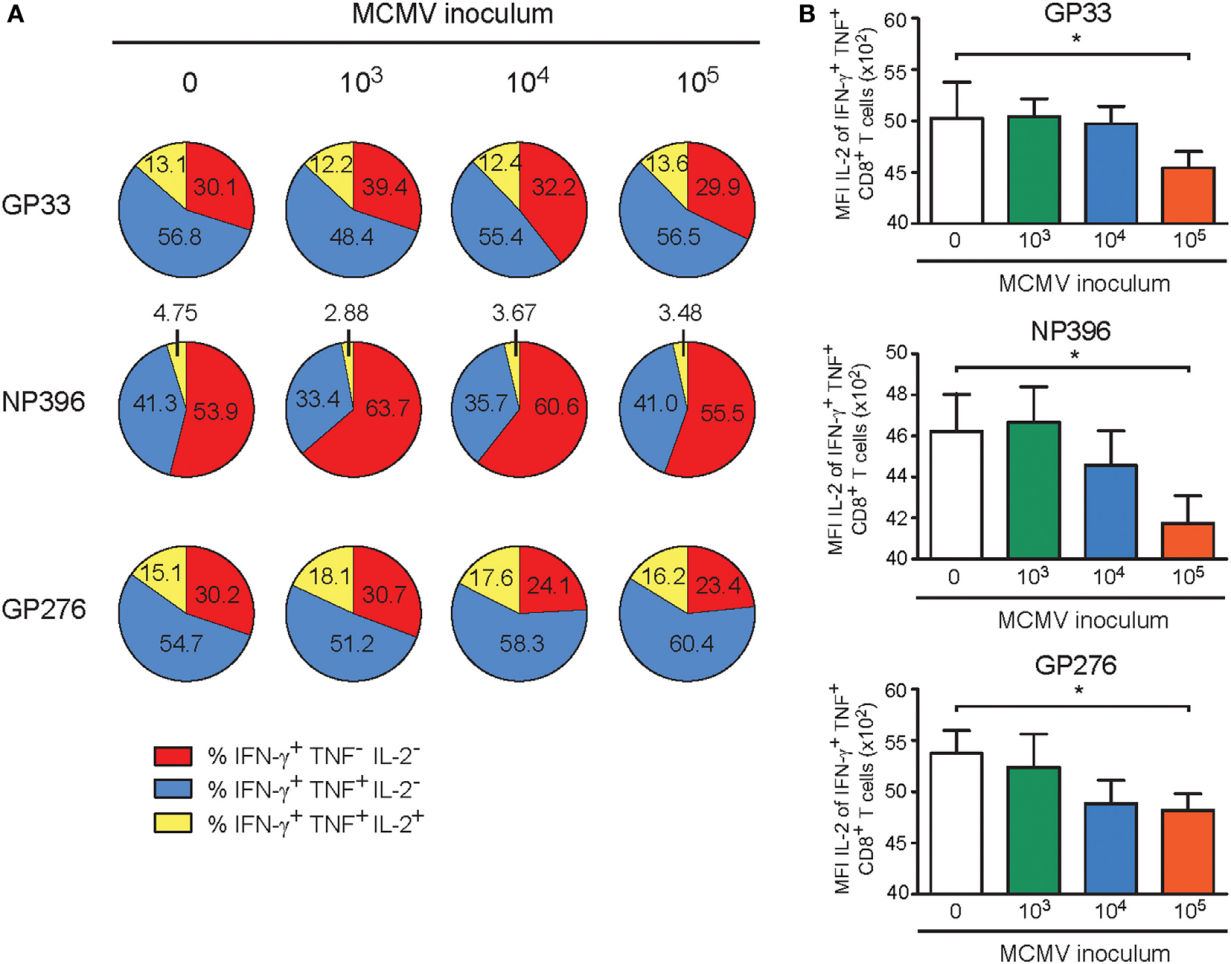
High-dose CMV infection impairs the autocrine IL-2 production of heterologous viral-specific T cells. Wild-type (WT) mice were kept uninfected or infected with 10^3^, 10^4^, or 10^5^ PFU mouse CMV (MCMV)-Smith, and at day 400 post-infection 8 mice per group were challenged with 2 × 10^5^ PFU lymphocytic choriomeningitis virus (LCMV)-Armstrong. At day 8 post LCMV infection, mice were analyzed and compared with uninfected littermate controls. **(A)** The cytokine polyfunctionality of splenic CD8^+^ T cells was determined after peptide restimulation. Pie charts depict the percentages of the single (IFN-γ), double (IFN-γ/TNF), and triple (IFN-γ/TNF/IL-2) cytokine producers of each antigen-specific T cell population upon peptide stimulation. **(B)** MFI of IL-2 expression of LCMV-specific CD8^+^ T cells. All data represents mean values + SEM (*n* = 8 per group, *P < 0.05). Data were pooled from two independent experiments.

Next, we aimed to determine if the MCMV dose-associated alterations could also impact the cytokine polyfunctionality of the LCMV-specific CD8^+^ T cell responses in the aged MCMV-infected mice after heterologous infection with LCMV. Following *ex vivo* stimulation with class I-restricted peptides GP_33–41_, NP_396–404_, and GP_276–286_, the frequencies of the single (IFN-γ), double (IFN-γ and TNF), and triple (IFN-γ, TNF, and IL-2) producing LCMV-specific CD8^+^ T cells were determined. Although an obvious altered cytokine profile was not detected (Figure 7A), the responding LCMV-specific T cells exhibited a lower IL-2 expression on a per cell basis in only those mice that were previously inoculated with the highest dose of MCMV (Figure 7B). Analysis of the CD4^+^ T cell response against the LCMV epitope GP_61–80_ showed that the cytokine polyfunctionality of the LCMV-specific CD4^+^ T cells are not affected by lifelong MCMV infection (Figure S4B in Supplementary Material).

Taken together, these results establish that persistent MCMV infection induced by a high inoculum dose is able to decrease the development of heterologous adaptive immune responses upon encountering a new pathogen.

### The Inflammatory Milieu and Antibody Levels upon Heterologous Infection Are Influenced by the Inoculum Dose of MCMV

**Figure 8:**
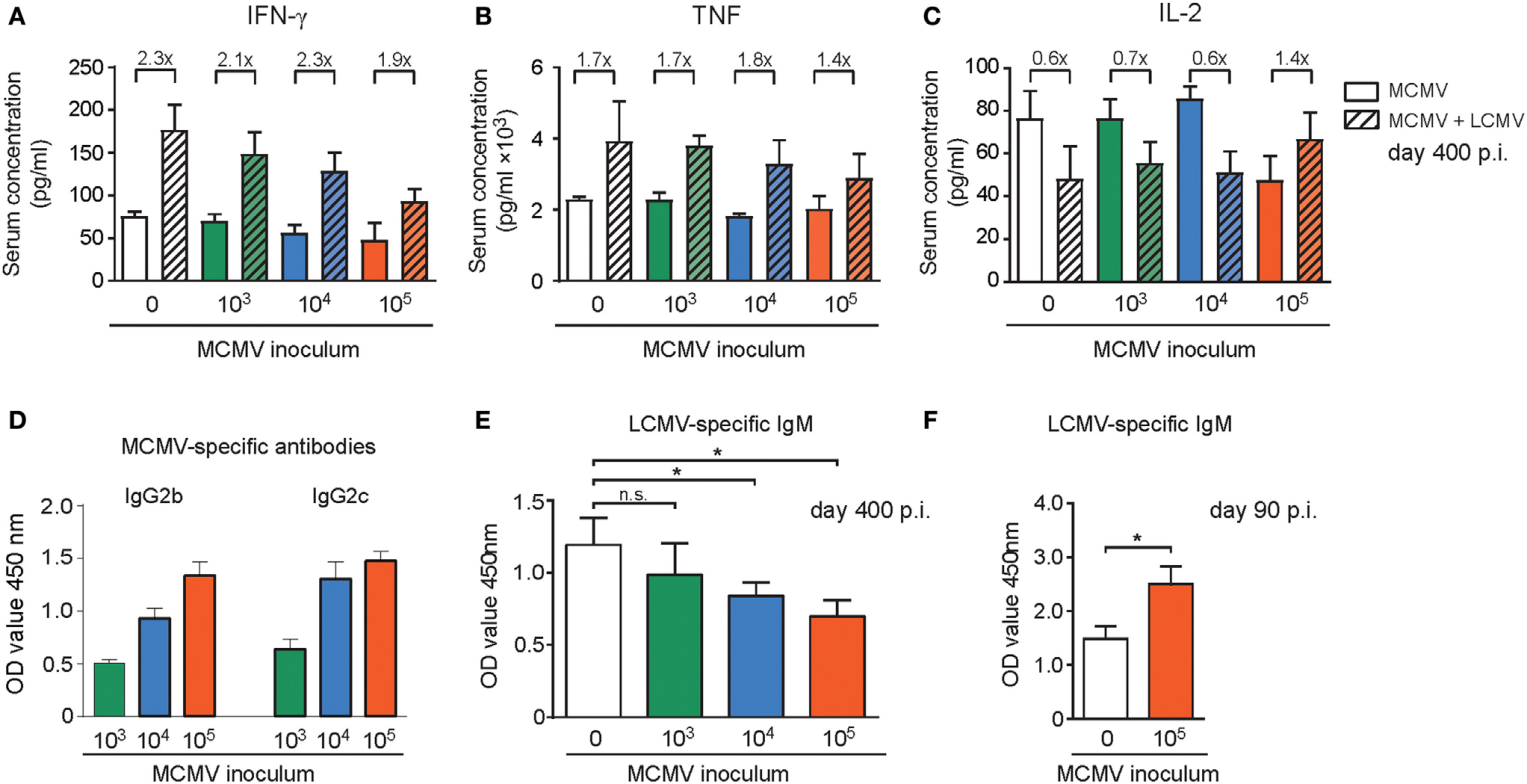
The inflammatory milieu and antibody levels upon heterologous infection are shaped by the inoculum dose of mouse CMV (MCMV). **(A–E)** Wild-type (WT) mice were kept uninfected or infected with 10^3^, 10^4^, or 10^5^ PFU MCMV-Smith (*n* = 16 mice per group), and at day 400 post-infection 8 mice per group were challenged with 2 × 10^5^ PFU lymphocytic choriomeningitis virus (LCMV)-Armstrong. At day 8 post LCMV infection, mice were analyzed and compared with uninfected littermate controls. Cytokines and chemokines in the serum were determined by mouse cytokine bio-plex immunoassays **(A–C)**, and MCMV **(D)** or LCMV-specific **(E,F)** antibody levels were determined by ELISA. **(A–C)** Shown are the serum concentrations of IFN-γ **(A)**, TNF **(B)**, and IL-2 **(D)**. MCMV-specific IgG antibody levels in LCMV unchallenged mice **(E)** Levels of LCMV-specific IgM antibodies in LCMV-challenged mice. **(F)** WT mice were kept uninfected or infected with 10^5^ PFU MCMV-Smith, and at day 90 post-infection mice were challenged with 2 × 10^5^ PFU LCMV-Armstrong. Blood was taken 8 days after LCMV infection and levels of LCMV-specific IgM antibodies within the serum were determined. Data represents mean values + SEM (*n* = 8 per group, *P < 0.05). Data were pooled from two independent experiments.

Chronic low grade inflammation accompanied with persistent CMV infection might affect antigen-presenting cells in such a way that the priming of T cells against newly encountered antigens is hampered. To test whether the size of the inoculum dose might influence low grade inflammation in steady state and upon heterologous infection, we determined cytokine and chemokine serum levels. Analysis of the tested cytokine and chemokine levels revealed no substantial changes between mice that were infected with different doses of MCMV at day 400 post MCMV infection (Figure S6 in Supplementary Material). Upon LCMV infection, we observed for a number of cytokines/chemokines a major increase, albeit mostly this was not influenced by the MCMV inoculum dose. However, a MCMV dose-dependent decrease in IFN-γ serum levels is apparent (Figure 8A). For the TNF serum levels the same trend was observed (Figure 8B). Analysis of the IL-2 serum concentration revealed that IL-2 levels were lower in the high MCMV dose inoculating mice (Figure 8C), which corresponds to the lower amounts of IL-2 produced by the inflationary T cells. In contrast to the other cytokines present in serum, upon LCMV infection the IL-2 levels decreased in the mice without prior MCMV infection and in the mice inoculated with 1 × 10^3^ and 1 × 10^4^ PFU. Whereas in mice that experienced lifelong infection with the highest MCMV dose, the IL-2 concentration increased, suggesting a lower rate of IL-2 consumption by the T cells (Figure 8C). Thus, the MCMV inoculum dose differentially shapes the inflammatory milieu with respect to effector T cell-associated cytokines (IFN-γ, TNF, IL-2) upon encountering a new pathogen.

Given the correlation between MCMV-specific antibody titers and the infectious dose (Figure 8D), we aimed to address whether the MCMV inoculum dosage particularly affects heterologous T cell responses or whether also heterologous B cell responses are influenced. In the MCMV-infected mice 8 days after LCMV infection the LCMV-specific IgM antibody induction was hampered and only in those mice that were previously inoculated with high-dose MCMV (Figure 8E). In young mice with and without a latent MCMV infection that were challenged with LCMV a reverse trend was observed. In these mice the LCMV-specific IgM production was increased in mice with a latent MCMV infection (Figure 8F). Thus, only in aged mice the MCMV inoculum size inversely correlates with LCMV-specific IgM induction after heterologous infection.

### High-Dose CMV Infection Hampers Control of Heterologous Infections and Correlates to the Phenotypical Alterations of Virus-Specific CD8^+^ T Cells

**Figure 9:**
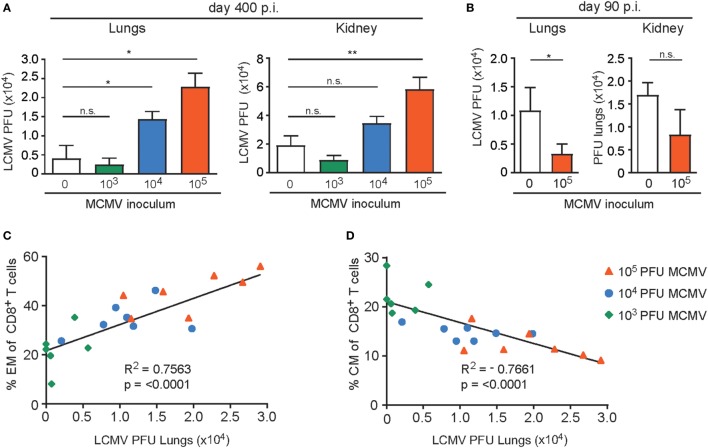
High-dose CMV infection hampers control of heterologous infections and correlates to the phenotypical alterations of virus-specific CD8^+^ T cells. **(A)** Wild-type (WT) mice were kept uninfected or infected with 10^3^, 10^4^, or 10^5^ PFU mouse CMV (MCMV)-Smith (*n* = 16 mice per group), and at day 400 post-infection 8 mice per group were challenged with 2 × 10^5^ PFU lymphocytic choriomeningitis virus (LCMV)-Armstrong. At day 8 post LCMV infection, mice were analyzed for the LCMV viral load in lungs and kidneys. **(B)** WT mice were kept uninfected or infected with or 10^5^ PFU MCMV-Smith (*n* = 8 mice per group), and at day 90 post-infection challenged with 2 × 10^5^ PFU LCMV-Armstrong. At day 8 post LCMV infection, mice were analyzed for the LCMV viral load. **(A,B)** All data represents mean values + SEM (*n* = 8 per group). n.s. *P* > 0.05; * *P* < 0.05; ** *P* < 0.01. **(C–D)** Correlation between lung LCMV titers and the frequency of peripheral effector-memory (EM) **(C)** and central-memory (CM) **(D)** CD8^+^ T cells 400 days post-infection of the same mice. Pearson’s correlations were used to determine the strength of the correlations ****P < 0.0001. Data were pooled from two independent experiments.

To determine if the aforementioned MCMV-induced immune alterations resulted in hampered LCMV control, we determined the LCMV viral load in the lungs and kidneys of the uninfected and MCMV-infected mice. We observed that compared to MCMV-naive mice the viral load was significantly increased in mice that were inoculated with 1 × 10^5^ PFU MCMV. Importantly, in mice that were inoculated with lower doses of MCMV, control of LCMV replication was not significantly affected (Figure 9A). Importantly in young mice inoculated with a high-dose, LCMV infection was even slightly better controlled (Figure 9B).

The frequency of EM T cells in peripheral blood of aged mice correlates with the inoculum dose while an inverse correlation is seen for CM CD8^+^ T cells. To determine whether the frequency of EM CD8^+^ T cells before challenge with LCMV also correlates with the ability to control heterologous infection, we plotted the EM frequencies of the total CD8^+^ T cell population against the LCMV viral load in the lungs. Clearly, a strong connection between the EM phenotype and LCMV titers in the lungs is observed (Figure 9C). A reverse correlation was found when the frequency of CM T cells was plotted (Figure 9D). These data indicate that the frequency of EM CD8^+^ T cells (or inversely the CM CD8^+^ T cells) can be used as predictors of a possible defective capacity of the host to respond to newly encountered pathogens.

## Discussion

The possible contribution of CMV to immune senescence has received considerable attention but remained controversial (20, 21). To extend our knowledge with respect to the role of CMV in immune senescence, we performed a highly controlled prospective study revealing that the size of the initial viral inoculum dictates the degree of CMV-induced immune alterations in long-lasting infection. Our data revealed a clear correlation between the magnitude of the CD8^+^ T cell response and the frequency of CMV-specific CD8^+^ T cells exhibiting an EM phenotype, which was apparent in CMV-infected mice and humans. Importantly, we show that solely a high CMV infectious dose results in immune perturbations that are substantial enough to impair heterologous anti-viral immunity, thereby providing a better understanding of CMV in immune senescence and in addition settles previous controversies in this respect.

In agreement with our results, several studies have shown that MCMV infection with a high infectious dose causes immune perturbations that impair CD8^+^ T cell immunity later in life (22–24). Previously, we have shown that the influence of CMV on eliciting inflationary EM-like CD8^+^ T cells populations is depending on the inoculum dose (27). In the current study, we have appreciated this differential impact of dissimilar CMV dosages and show that CMV infection does not by definition impairs immunity, but specifically a high infectious dose is a prerequisite for CMV-associated immune senescence. Conceivably, in immune compromised hosts where viral control is hampered, already a low viral dose might result in CMV-related immune senescence. Although we have shown that weekly reinfection with the same dose does not increase EM T cell accumulation, it should be noted that in addition to the initial infectious dose, the CMV dosage during reinfection and the level of pre-existing CMV-specific neutralizing antibodies could impact the degree of CD8^+^ T cell accumulation. We assume that in humans, where reinfection is known to occur (39), the viral dose differs in subsequent reinfection. We consider it likely that CMV reinfection with increasing infectious doses does intensify the degree of EM T cell accumulation and, hence, thereby possibly immune senescence.

In human individuals, correlations between HCMV positivity and impaired immunity are controversial since this relationship is observed in some but not all studies (12–18, 40–44). Our results can at least partly explain this controversy, because it is highly conceivable that in humans CMV infection occurs with infectious dosages that are highly variable. In addition, besides the infectious dose also host intrinsic factors such as genetic predisposition can impact the CMV grade, and hence, contribute possibly to immune senescence.

The accumulation of CMV-specific EM CD8^+^ T cells, which correlate with impaired heterologous immunity, might by itself be (partly) causative for the negative impact of long-standing latent CMV on heterologous immunity. In case of high-dose infection, the frequency of CMV-specific CD8^+^ T cells might reach a certain threshold resulting in diminished priming of naive T cells against newly encountered pathogens. Indeed, detailed cluster analysis of the EM development of the circulating CMV-specific CD8^+^ T cells showed that high-dose infection causes a differentiation pathway that progresses faster throughout the life span of the host, suggesting a virus–host equilibrium that is not fixed and apparently is influenced by aging and the infectious dose. Whether ongoing EM differentiation takes also place in tissues requires further investigations. A higher infectious dose is known to result in a higher level of reactivation from latency (45) and this is in line with the observation that a higher CMV dose results in larger reservoirs of latent CMV (27). As a result, stimulation of CMV-specific T cells is increased, thereby accelerating differentiation. Moreover, we observed that the LCMV-specific CD8^+^ T cells in mice infected with a high CMV dose are not properly primed as evidenced by decreased upregulation of CD27 and lower IL-2 production. It is considered that in aged individuals priming occurs less as compared to young (46). The additional amounts of (activated) T cells might further diminish this by competing for T cell growth factors or even by competition at the APC level. Nonetheless, we consider it likely that the accumulation of CMV-specific T cells is not solely responsible for impairing immune responses against newly encountered pathogens. For example, our analysis also revealed a weakened LCMV-specific B cell response in those mice that were infected with the highest dose of MCMV. In mouse and human CMV infection, accumulation of CMV-specific antibodies has been reported (27, 47, 48). This may indicate that also accumulation of CMV-specific antibody-producing B cells occurs. Possibly these CMV-specific plasma cells compete with B cells that are specific for the newly encountered pathogen.

In contrast to what we observed in aged mice, in young mice CMV infection seems to improve immunity against other pathogens, which is in agreement with other reports showing beneficial effects of herpesvirus infection on protection from other infections (18, 49). However, the positive effects of herpesvirus infections in young mice is transient (18, 49, 50), which may be due to a temporary effect on both the innate and adaptive immune system. In addition, Furman et al. showed that CMV-seropositive young adults displayed an increase in CD8^+^ T cell sensitivity and an improvement in the antibody response upon influenza vaccination, and it was shown that IFN-γ was required for the CMV-associated improved cross-protection (18). Upon heterologous challenge with LCMV, we observed that serum levels of IFN-γ were significantly reduced in mice that were infected with the highest dose of CMV compared to mice that were CMV negative. This suggests that a “fit” immune system in which IFN-γ production is properly induced is crucial for the CMV-associated enhancement of heterologous immune responses. We consider it likely that aging by itself abolishes the positive effect of CMV by decreasing the IFN-γ-producing capacity and that a combination of old age and extensive CMV-induced immune aberrations shift the balance toward a negative effect of CMV on heterologous immunity.

In summary, our results demonstrate that the infectious dose of CMV is a key determinant for the immunological outcome and consequently, also projects the possible impact for heterologous immunity later in life. Importantly, here we show for the first time that only infection with a high CMV infectious dose impairs the immune response against newly encountered pathogens in aged hosts. Thus, reduction of the latent/lytic viral load (by anti-viral drugs or vaccination) can be beneficial to diminish CMV-associated immune senescence. Future prospective longitudinal studies that incorporate stratification based on the strength of the HCMV-specific immune response are required to further delineate the contribution of CMV to immune senescence.

## Ethics Statement

The inclusion of patients has been conducted in accordance with the ethical principles set out in the Declaration of Helsinki. Both patient inclusion and blood sample collection were done with approval of the Amsterdam Medical Center Medical Ethical Committee. Written informed consent was obtained prior to data collection.

All animal experiments were approved by the Animal Experiments Committee of the Leiden University Medical Center (LUMC) and performed according to the Dutch Experiments on Animals Act that serves the implementation of “Guidelines on the protection of experimental animals” by the Council of Europe and the guide to animal experimentation set by the LUMC.

## Author Contributions

AR designed and performed most of the experiments and data analysis, and wrote the manuscript. ER performed and analyzed the flow cytometry experiments of HCMV seropositive individuals, and reviewed the manuscript. EG and SW conducted experiments, assisted with flow cytometry and reviewed the manuscript. TH supported the Cytosplore analysis and reviewed the manuscript. FK supported the Cytosplore analysis and reviewed the manuscript. LC-S and JN-Ž performed some of the experiments and reviewed the manuscript. IB and RL reviewed the manuscript. VU supported the Cytosplore analysis and reviewed the manuscript. RA designed and supervised the study, wrote the manuscript, and provided major funding for the study.

## Conflict of Interest Statement

The authors declare that the research was conducted in the absence of any commercial or financial relationships that could be construed as a potential conflict of interest.
