# Supplementary material for: The Contribution of Cytomegalovirus Infection to Immune Senescence Is Set by the Infectious Dose
Source: Front Immunol. 2018 Jan 10;8:1953. doi: 10.3389/fimmu.2017.01953 (PMC5768196; doi:10.3389/fimmu.2017.01953)
Supplement: Supplementary file 1 [file Data_Sheet_1.PDF]

Supplementary Material

The contribution of cytomegalovirus infection to immune senescence is set by the infectious dose

Anke Redeker[1], Ester B.M. Remmerswaal[2,3¶], Esmé T.I. van der Gracht[1¶], Suzanne P. M. Welten[1,#], Thomas Höllt[4], Frits Koning[1], Luka Cicin-Sain[5], Janko Nikolich-Žugich[6], Ineke J.M. ten Berge[2,3], René A.W. van Lier[7], Vincent van Unen[1], Ramon Arens[1]

*** Correspondence:** R.Arens@lumc.nl

Supplementary Figures:

Supplementary Figure S1. TCR Vβ usage and phenotype of CD8 T cells in MCMV infection.

Supplementary Figure S2. Ongoing effector-memory CD8+ T cell differentiation upon CMV infection.

Supplementary Figure S3. Increased effector-memory CD8+ T cell differentiation in high dose CMV infection.

Supplementary Figure S4. MCMV and LCMV-specific CD4+ T cell responses.

Supplementary Figure S5. Expression of activation markers on LCMV-specific CD8+ T cells.

Supplementary Figure S6. Cytokine and chemokine serum concentration.

Supplementary figure S1. Disparate effects of CMV infection on $CD8^+$ and $CD4^+$ T cell subsets.

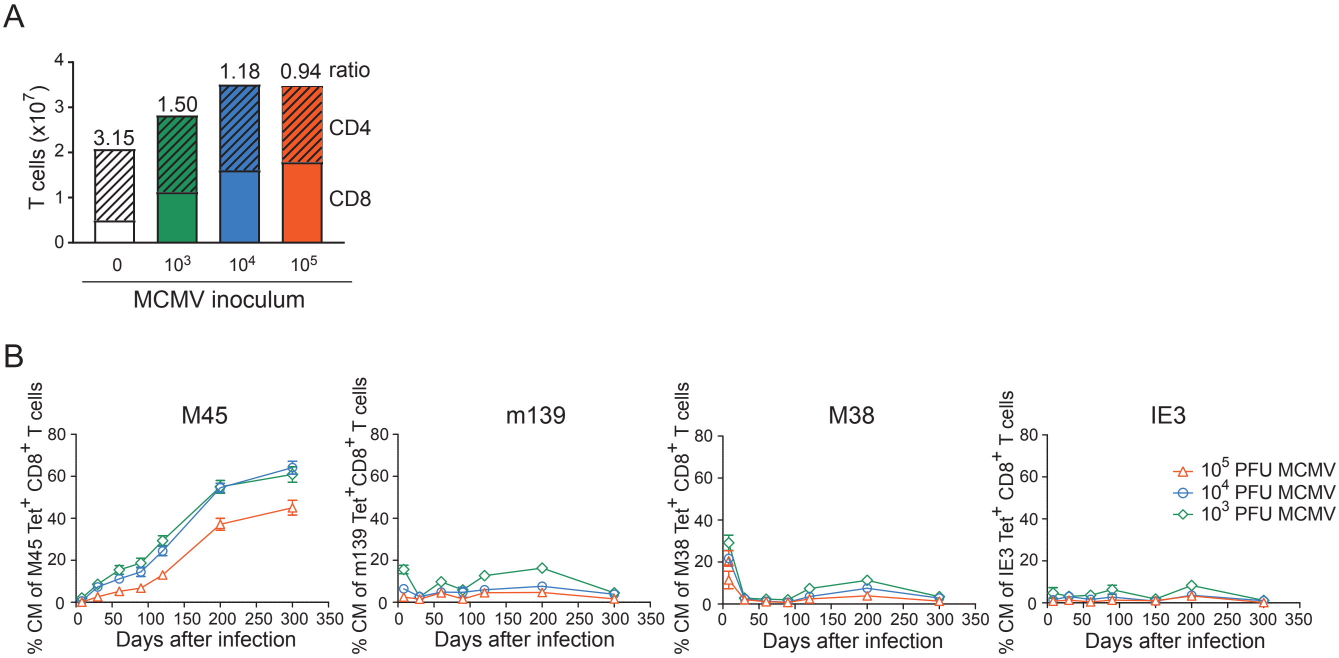

Disparate effects of CMV infection on $CD8^+$ and $CD4^+$ T cell subsets. WT mice were infected with 10^3, 10^4 or 10^5 PFU MCMV-Smith. (A) Absolute counts of the total $CD8^+$ and $CD4^+$ T cell populations in spleen at day 400 post-infection. The CD4/CD8 T cell ratio is specified. (B) Wild-type (WT) mice were infected with 10^3, 10^4 or 10^5 PFU MCMV-Smith (n=16 mice per group). Graphs depict the average frequencies of central-memory ($CD44^{high}CD62L^+KLRG1^-$) type $CD8^+$ T cells within the MCMV-specific $CD8^+$ T cell populations in blood. All data represents mean values (n = 16 per group).

Supplementary figure S2. Increased effector-memory $CD8^+$ T cell differentiation in high dose CMV infection.

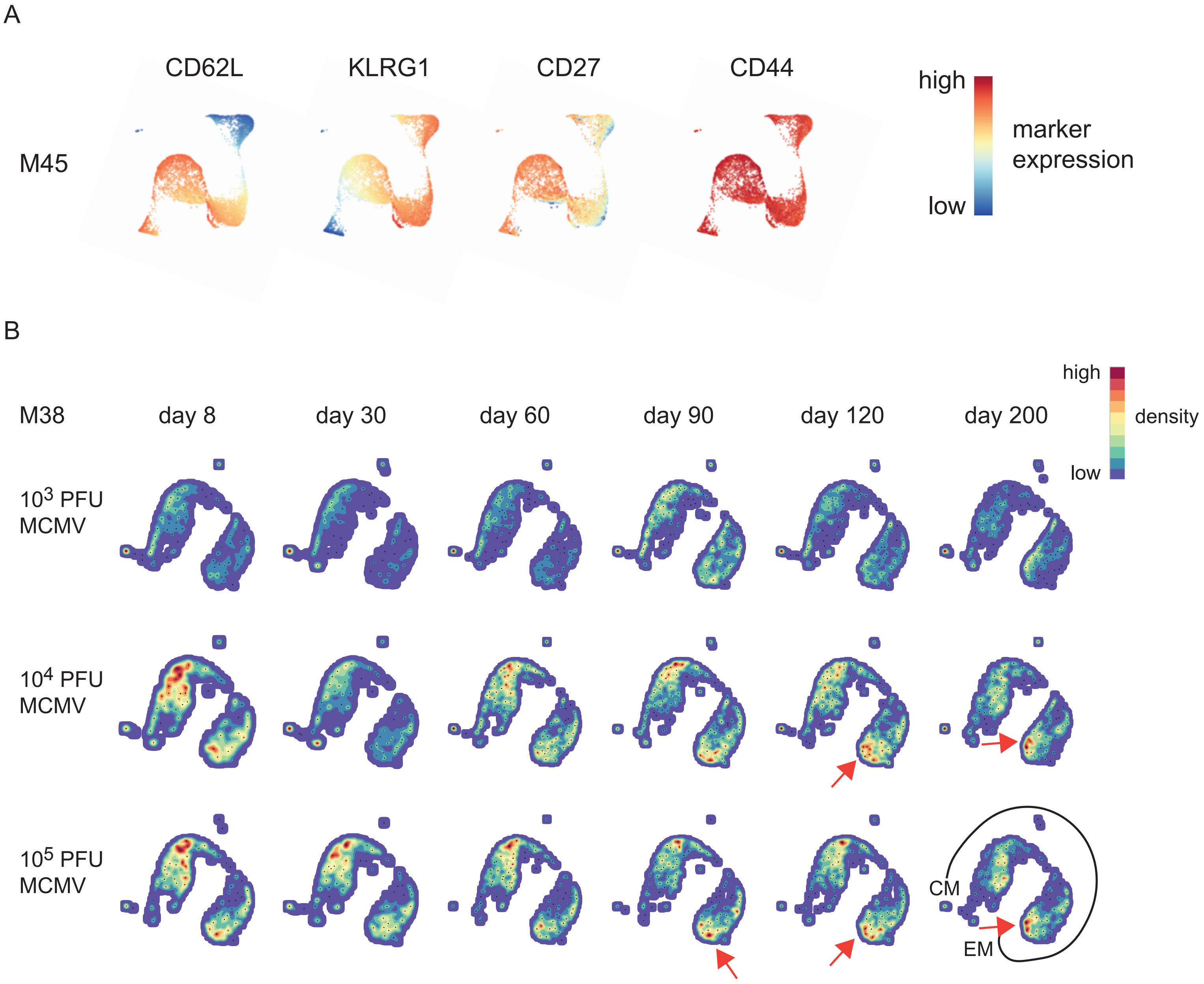

Increased effector-memory CD8+ T cell differentiation in high dose CMV infection. WT mice were infected with 10^3, 10^4 or 10^5 PFU MCMV-Smith. MCMV-specific $CD8^+$ T cells (i.e., specific to epitopes derived from the MCMV proteins M45, m139, M38 and IE3) in blood were stained with MHC class I tetramers combined with cell surface markers (CD62L, KLRG1, CD27 and CD44). (A) A-tSNE plots visualize the intensity of single marker expression as a scatterplot. (B) A-tSNE plots depict the phenotype of the M38-specific $CD8^+$ T cell response of the 10^3, 10^4 and 10^5 PFU MCMV-infected mice for each time point after infection (n=16 mice per MCMV dose). Data were pooled from two independent experiments. In the A-tSNE plots the differentiation path from the CM and EM phenotype is specified. The red arrows indicate the ongoing shift toward a higher advanced EM-phenotype.

Supplementary figure S3. Increased effector-memory CD8$^+$ T cell differentiation in high dose CMV infection.

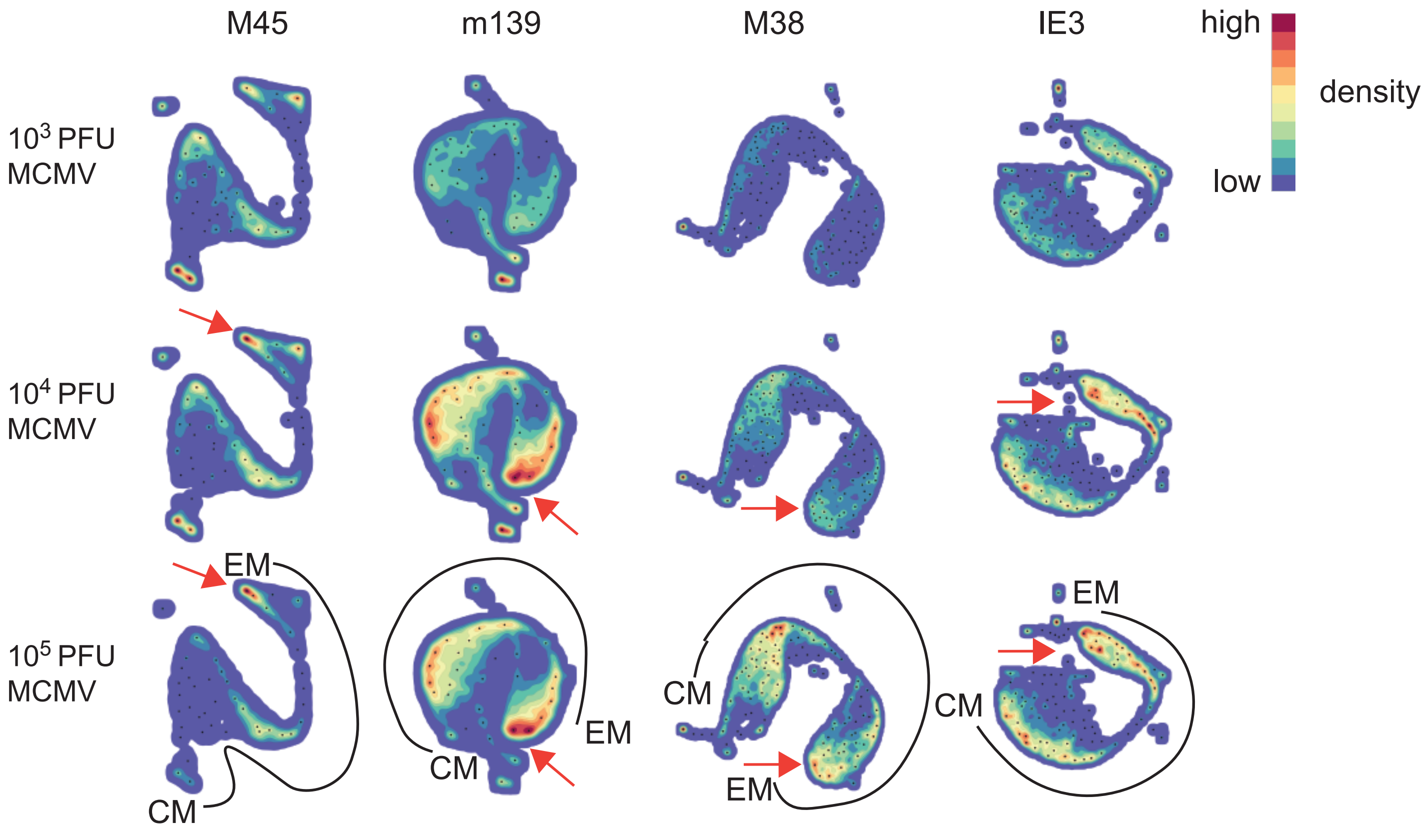

Increased effector-memory CD8+ T cell differentiation in high dose CMV infection. WT mice were infected with 10^3, 10^4 or 10^5 PFU MCMV-Smith. MCMV-specific CD8$^+$ T cells (i.e., specific to epitopes derived from the MCMV proteins M45, m139, M38 and IE3) in blood were stained with MHC class I tetramers combined with cell surface markers (CD62L, KLRG1, CD27 and CD44). Cytosplore analysis of the MCMV-specific CD8$^+$ T cells in time. A-tSNE plots depict the pooled phenotypic data of MCMV-specific CD8$^+$ T cell responses of each time point after infection of the 10^3, 10^4 and 10^5 PFU MCMV-infected mice. In the A-tSNE plots the differentiation path from the CM and EM phenotype is specified. The red arrows indicate the ongoing shift toward a higher advanced EM-phenotype.

Supplementary figure S3. MCMV and LCMV-specific CD4$^+$ T cell responses.

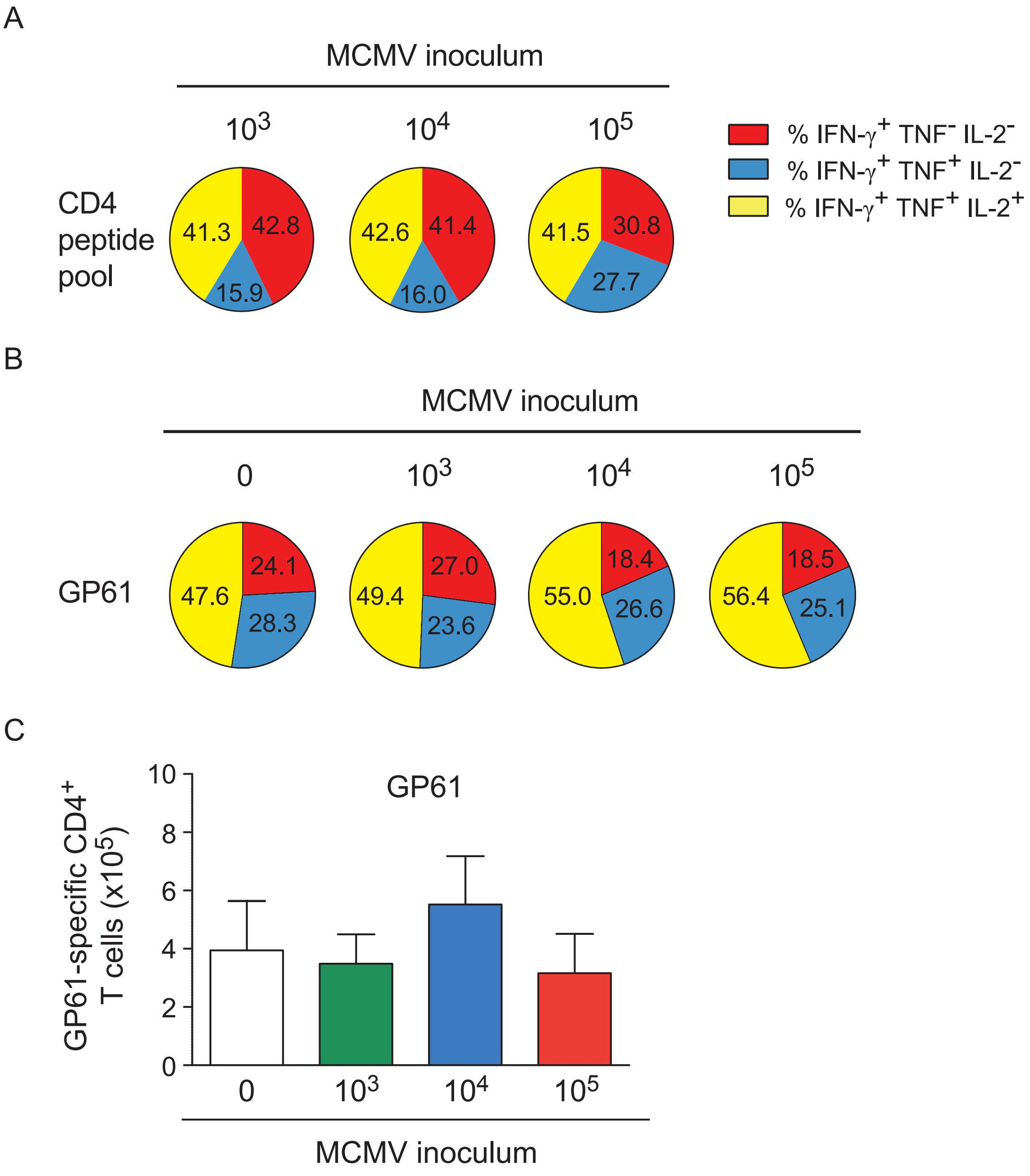

MCMV and LCMV-specific CD4$^+$ T cell responses. WT mice were kept uninfected or infected with 10^3, 10^4 or 10^5 PFU MCMV-Smith (n=16 mice per group), and at day 400 post-infection 8 mice per group were challenged with 2×10^5 PFU LCMV-Armstrong. (A) The cytokine polyfunctionality of MCMV-specific splenic CD4$^+$ T cells was determined after peptide restimulation at day 400 post-infection (of the LCMV-unchallenged group). (B) The cytokine polyfunctionality of LCMV-specific splenic CD4$^+$ T cells was determined after peptide restimulation at day 400 post-infection (of the LCMV-challenged group). Pie charts depict the percentages of the single (IFN-γ), double (IFN-γ/TNF) and triple (IFN-γ/TNF/IL-2) cytokine producers upon peptide stimulation with (A) a pool of class II-restricted MCMV peptides or (B) GP61 peptide. (C) Absolute numbers of GP61-specific CD4$^+$ T cells as determined by IFN-γ production. Data represents mean values (n = 8 per group).

Supplementary figure S4. MCMV and LCMV-specific CD4$^+$ T cell responses.

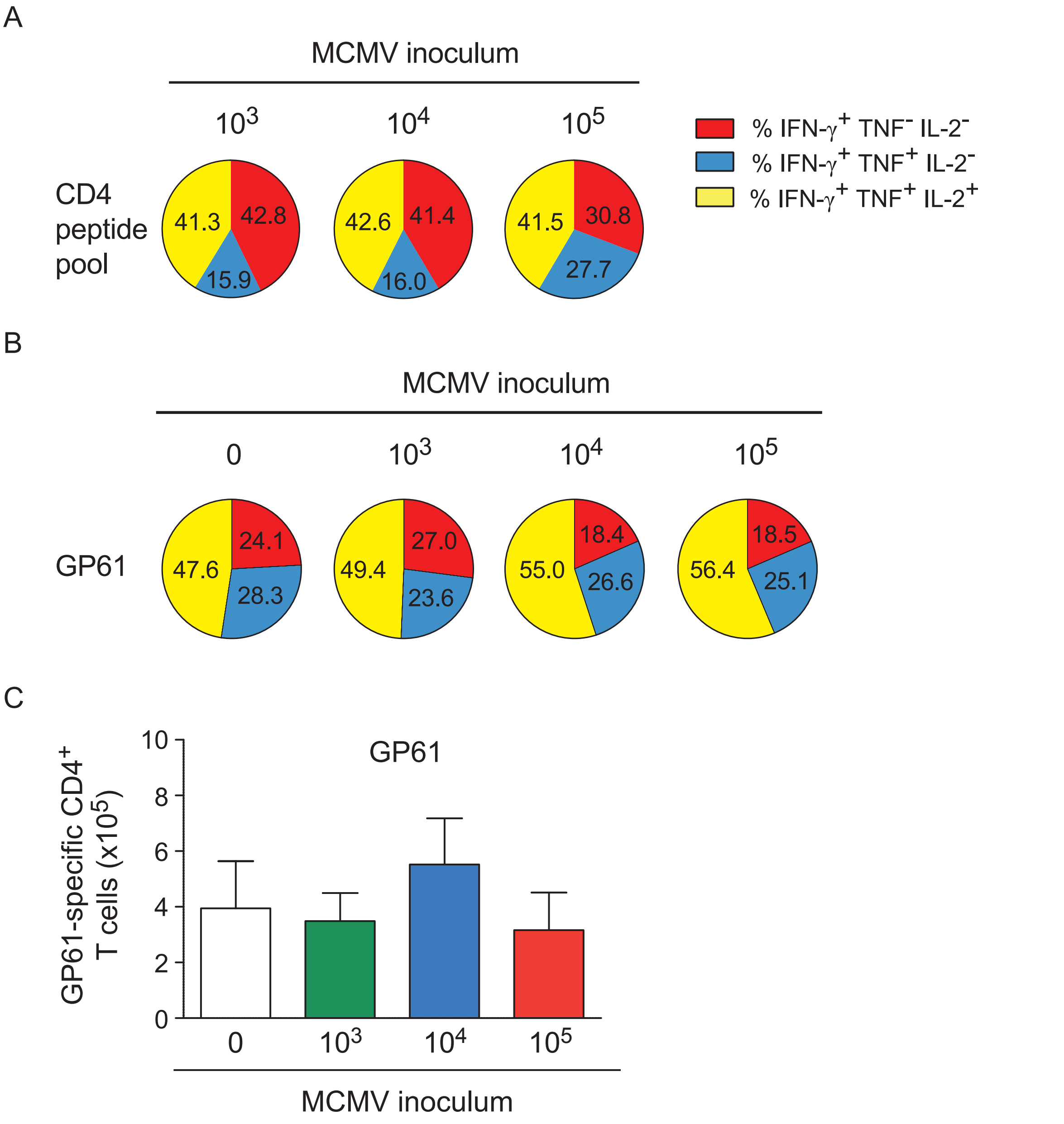

MCMV and LCMV-specific CD4$^+$ T cell responses. WT mice were kept uninfected or infected with 10^3, 10^4 or 10^5 PFU MCMV-Smith (n=16 mice per group), and at day 400 post-infection 8 mice per group were challenged with 2×10^5 PFU LCMV-Armstrong. (A) The cytokine polyfunctionality of MCMV-specific splenic CD4$^+$ T cells was determined after peptide restimulation at day 400 post-infection (of the LCMV-unchallenged group). (B) The cytokine polyfunctionality of LCMV-specific splenic CD4$^+$ T cells was determined after peptide restimulation at day 400 post-infection (of the LCMV-challenged group). Pie charts depict the percentages of the single (IFN-γ), double (IFN-γ/TNF) and triple (IFN-γ/TNF/IL-2) cytokine producers upon peptide stimulation with (A) a pool of class II-restricted MCMV peptides or (B) GP61 peptide. (C) Absolute numbers of GP61-specific CD4$^+$ T cells as determined by IFN-γ production. Data represents mean values (n = 8 per group).

Supplemental figure S5. Expression of activation markers on LCMV-specific $CD8^+$ T cells.

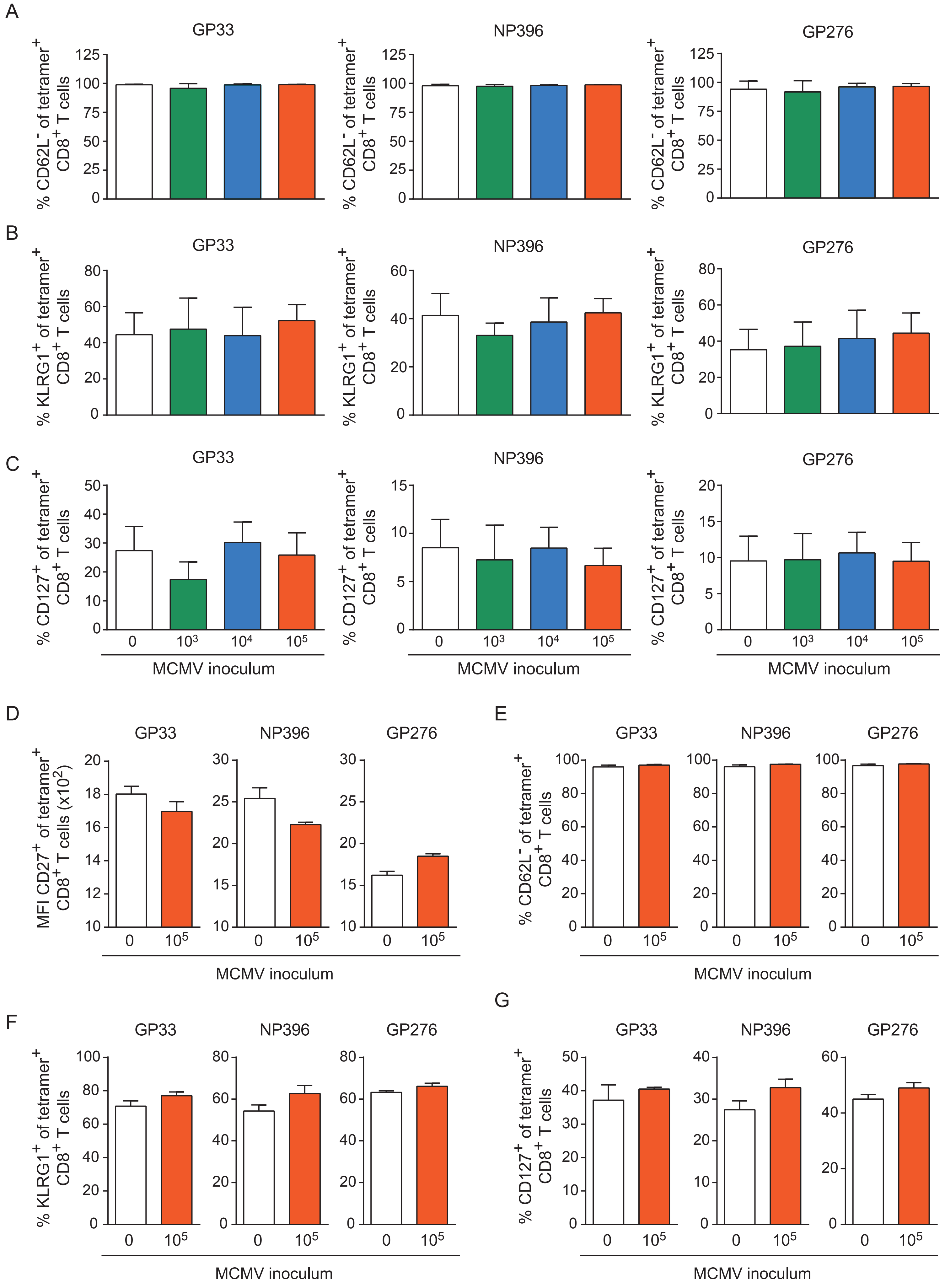

Expression of activation markers on LCMV-specific $CD8^+$ T cells. (A-C) WT mice were kept uninfected or infected with 10^3, 10^4 or 10^5 PFU MCMV-Smith (n=16 mice per group), and at day 400 post-infection 8 mice per group were challenged with 2×10^5 PFU LCMV-Armstrong. At day 8 post LCMV infection, mice were analyzed and compared with uninfected littermate controls. (A) Percentage of LCMV-specific $CD8^+$ T cells that are $CD62^{low}$. (B-C) Percentage of LCMV-specific $CD8^+$ T cells expressing (B) KLRG1 or (C) CD127. (D-G) WT mice were kept uninfected or infected with or 10^5 PFU MCMV-Smith (n=8 mice per group), and at day 90 post-infection challenged with 2×10^5 PFU LCMV-Armstrong. At day 8 post LCMV infection, mice were analyzed and compared with uninfected littermate controls. (D) Mean fluorescence intensity (MFI) of CD27 expression on LCMV-specific $CD8^+$ T cells. (E) Percentage of LCMV-specific $CD8^+$ T cells that are CD62Llow. (F-G) Percentage of LCMV-specific $CD8^+$ T cells expressing (F) KLRG1 or (G) CD127.

Supplemental figure S6. Cytokine and chemokine serum concentration.

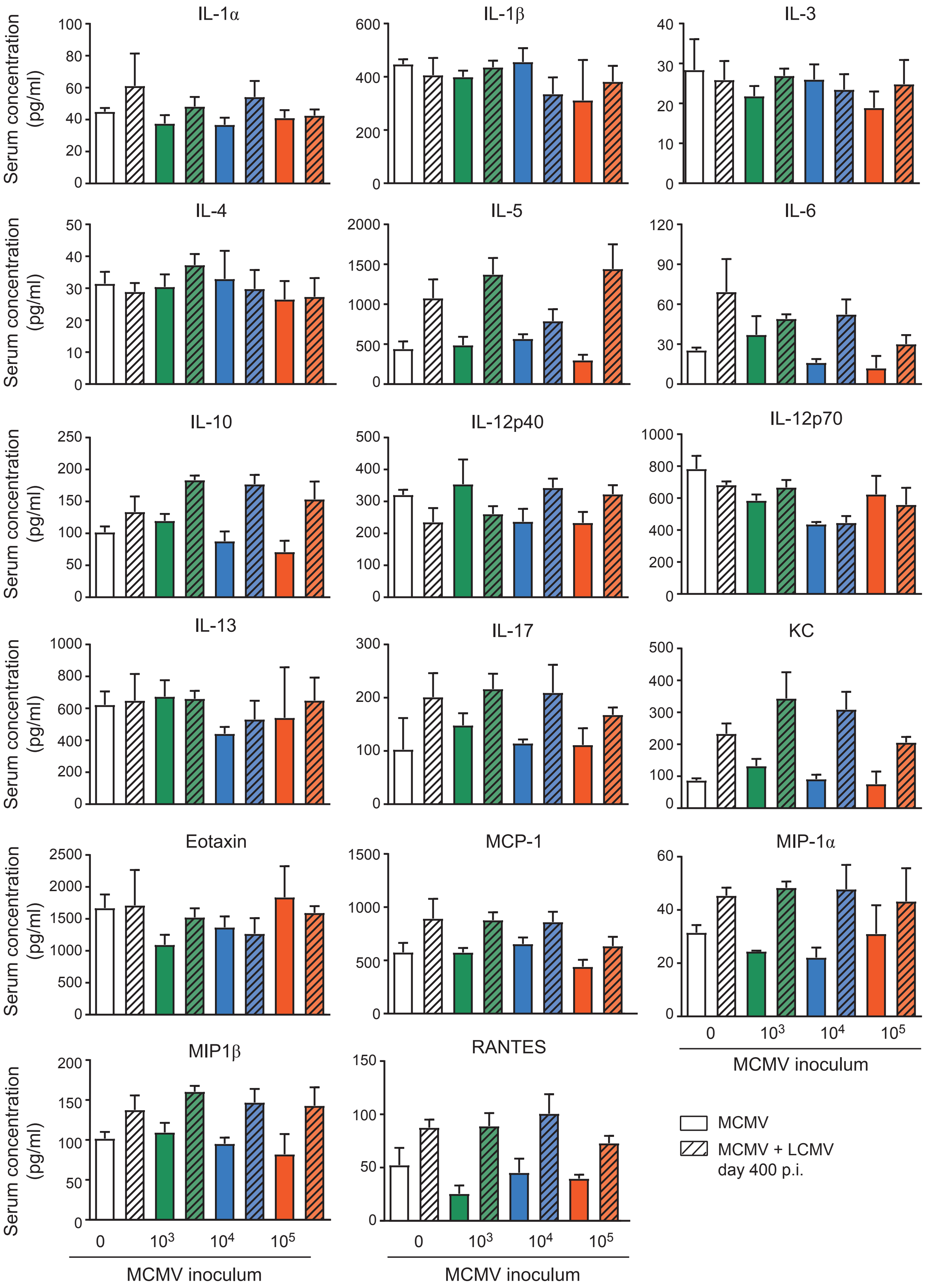

Cytokine and chemokine serum concentration. WT mice were kept uninfected or infected with 10^3, 10^4 or 10^5 PFU MCMV-Smith (n=16 mice per group), and at day 400 post-infection 8 mice per group were challenged with 2×10^5 PFU LCMV-Armstrong. At day 8 post LCMV infection, mice were analyzed and compared with uninfected littermate controls. Blood serum was taken and cytokine and chemokine concentration were determined by mouse cytokine bio-plex immunoassays. Shown are the serum concentrations of IL-1α, IL-1β, IL-3, IL-4, IL-5, IL-6, IL-10, IL12p40, IL12p70, IL-13, IL-17, KC, Eotaxin, MCP-1, MIP-1α, MIP-1β and RANTES.
